# A case of complete remission of Hodgkin lymphoma confirmed histopathologically by neck dissection

**DOI:** 10.1002/cnr2.1838

**Published:** 2023-05-31

**Authors:** Yu Ohashi, Kiyoto Shiga, Katsunori Katagiri, Daisuke Saito, Shin‐ichi Oikawa, Kodai Tsuchida, Jun Miyaguchi, Takahiro Kusaka, Hiroyuki Yamada

**Affiliations:** ^1^ Division of Oral and Maxillofacial Surgery, Department of Reconstructive Oral and Maxillofacial Surgery Iwate Medical University, School of Dentistry Morioka Iwate Japan; ^2^ Head and Neck Cancer Center Iwate Medical University Hospital Yahaba Iwate Japan; ^3^ Department of Otolaryngology‐Head and Neck Surgery Iwate Medical University, School of Medicine Yahaba Iwate Japan

**Keywords:** Hodgkin lymphoma, lymph node, neck metastasis, tongue cancer

## Abstract

**Background:**

Hodgkin lymphoma (HL) is diagnosed definitively by biopsy, and treatment is based on stage. Owing to the nature of the disease, post‐treatment efficacy is determined mainly by fluorodeoxyglucose‐positron emission tomography/computed tomography, and the efficacy of treatment is not confirmed by histopathology. We report a case of tongue cancer after treatment for HL, in which a post‐treatment lymph node with complete remission was histopathologically confirmed by neck dissection.

**Case:**

The patient was a 74‐year‐old man who was referred to our hospital for cancer on the right side of his tongue. He had previously undergone chemotherapy for HL involving the right side of his neck and achieved complete remission. Because he had cT3N2cM0 tongue cancer, glossectomy and bilateral neck dissection were performed. Surprisingly, histopathological examination revealed that there was neither metastatic lymph nodes nor lymphoma cells in his right neck. Moreover, there was no lymphatic structure in his remnant lymph nodes.

**Conclusion:**

This was a rare case in which complete remission of HL was confirmed by histopathological analysis. The absence of lymph node structure and lymphatic flow led to contralateral neck lymph node metastases of tongue cancer.

## 1 INTRODUCTION

Hodgkin lymphoma (HL) is a malignant disease of the lymphatic system. Overall survival rates for HL vary by age, race, and stage, but generally, the reported 5‐ and 10‐year survival rates are 83.0% and 78.1%, respectively.^1^ HL is diagnosed definitively by biopsy, and treatment is based on stage. Owing to the nature of the disease, post‐treatment efficacy is determined mainly by fluorodeoxyglucose‐positron emission tomography/computed tomography (FDG‐PET/CT),^2^ and the efficacy of treatment is not confirmed by histopathology. Therefore, the pathological findings of lymph node involvement after treatment of HL are unclear, and no related reports have been published, to our knowledge. There are no reports showing the pathological structure of HL that has gone into remission with treatment, and there is almost no opportunity for pathological confirmation of HL because of the nature of the disease and treatment. We report a rare case of complete remission in HL, as confirmed histopathologically by neck dissection.

## 2 CASE REPORT

**FIGURE 1:**
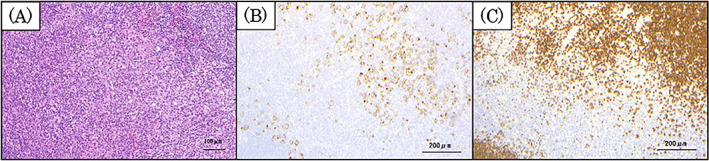
Histopathological findings of Hodgkin lymphoma with previous chemotherapy. Hematoxylin–eosin (H–E) staining of the lymph nodes showing solitary or small clusters of atypical cells with rather large, irregular nuclei in the paracortical region (A: ×200). Immunohistologically, the atypical cells were CD30‐positive (B: ×100). Strong positivity for PAX5 is also seen in the background B‐cells, with weak positivity in the tumor cells (C: ×100). CD30, cluster of differentiation 30.

**FIGURE 2:**
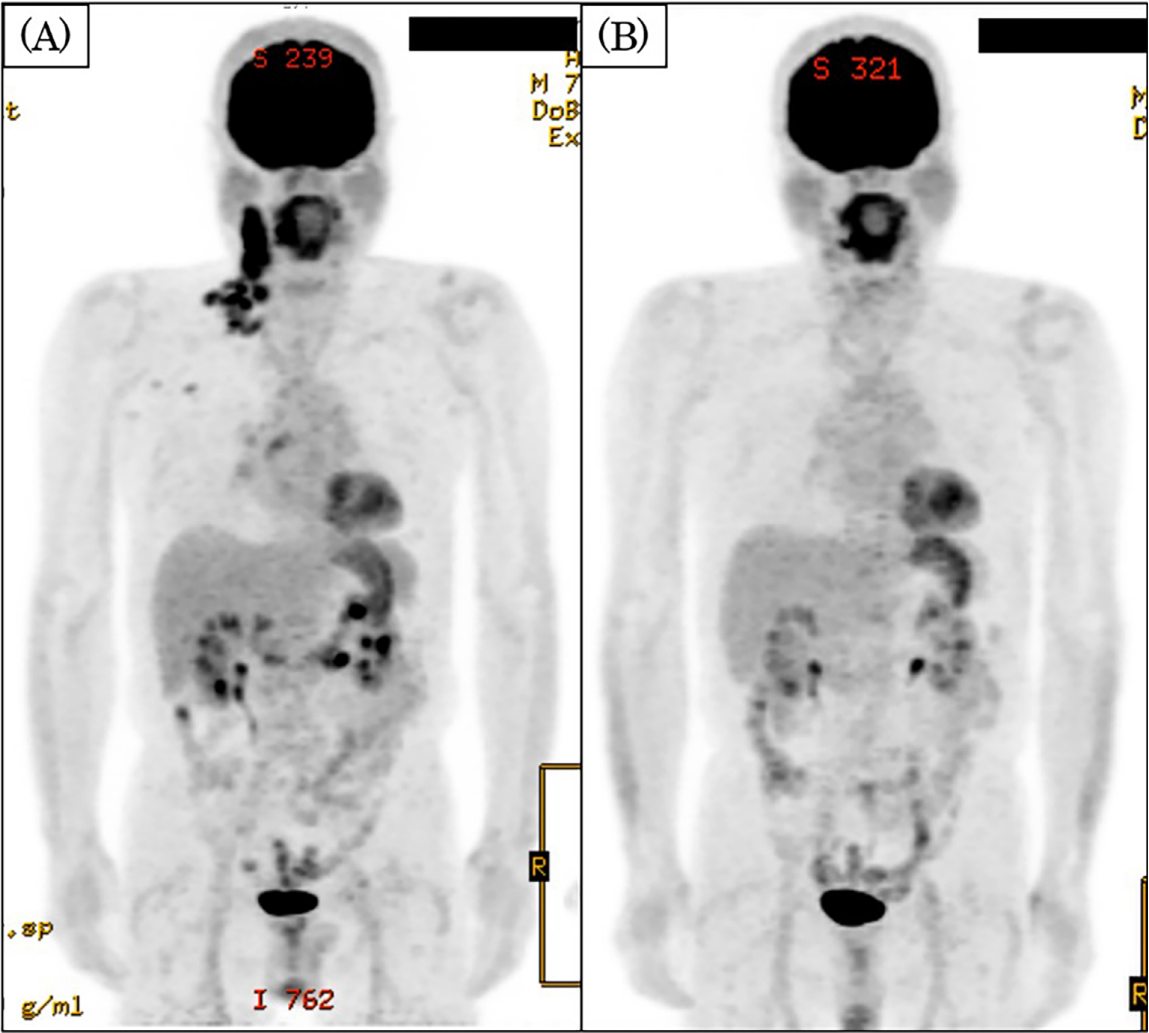
Fluorodeoxyglucose‐positron emission tomography/computed tomography (FDG‐PET/CT) findings before and after HL treatment. The HL‐affected areas were the right neck, right supraclavicular fossa, right pulmonary apex, right pulmonary hilum, right fifth posterior rib, and mediastinum. The image of the right neck shows abnormal accumulation of FDG with a maximum standardized uptake value (SUV_max_) of 15.7. The mediastinum shows abnormal accumulation with an SUV_max_ of 3.4 (A). HL was in complete remission (B). HL, Hodgkin lymphoma.

**FIGURE 3:**
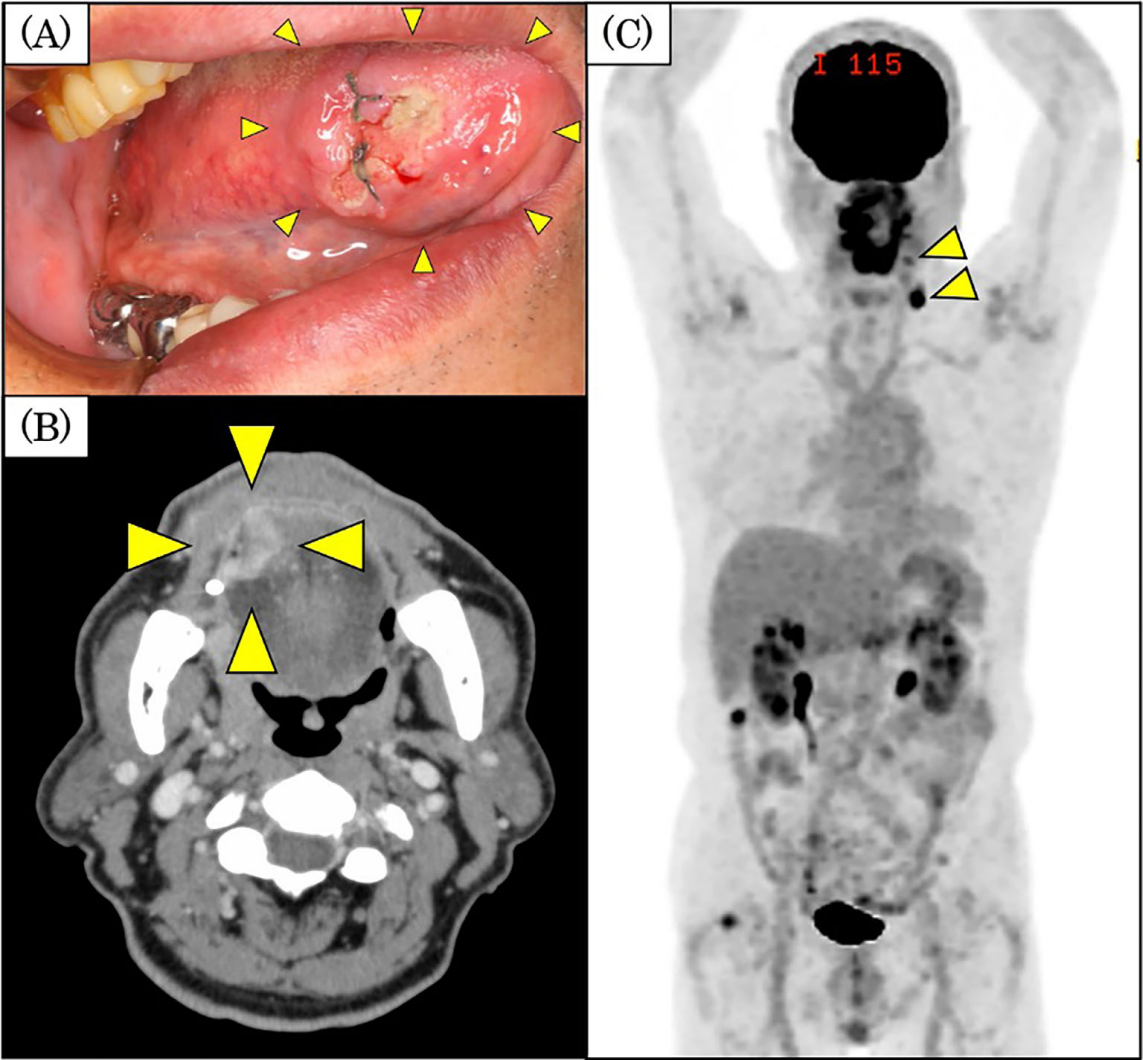
Findings of the tongue tumor and imaging findings. Intra‐oral findings (A). A tumor is visible on the right side of the tongue. The tumor was 28 mm in its longest diameter, with induration around the tumor and in the deep tissues. CT findings (B). The tumor measured 24.0 × 19.3 × 14.2 mm from the right lingual apex to the edge of the tongue. FDG‐PET/CT findings (C). The cervical lymph nodes are visible at left levels IB and III (arrowheads), with abnormal accumulation and SUV_max_ values of 4.10 and 9.00, respectively. There was no uptake in the lymph nodes in the right neck. CT, computed tomography; FDG‐PET/CT, fluorodeoxyglucose‐positron emission tomography/CT; SUV_max_, standardized maximum uptake value.

The patient was a 74‐year‐old man. He was referred to the Head and Neck Cancer Center, Iwate Medical University Hospital, in May 2021, because tongue cancer was suspected. He developed HL 1 year and 5 months prior to his first visit to our center. The pathological findings of HL were as follows: hematoxylin–eosin staining of the lymph nodes showed solitary or small clusters of atypical cells with rather large, irregular nuclei in the paracortical region (Figure 1A). Immunohistologically, the atypical cells were cluster of differentiation 30‐positive. PAX5 also showed strong positivity for background B‐cells and weak positivity for tumor cells (Figure 1B,C). The HL‐affected areas were the right neck, right supraclavicular fossa, right pulmonary apex, right pulmonary hilum, around the right posterior rib, and mediastinum (Figure 2A). The HL was the mixed cell type, stage II. The patient had received six courses of doxorubicin hydrochloride, bleomycin, vinblastine, and dacarbazine for HL 1 year and 6 months prior to presentation. The total dosage of each chemotherapeutic agent was 377.5 mg for doxorubicin hydrochloride, 85 mg for bleomycin, 90 mg for vinblastine, and 3360 mg for dacarbazine.^2^ Chemotherapy resulted in complete remission (Figure 2B). Blood examination at the initial visit to our center did not identify abnormal findings (Table 1). A tumor with ulceration and surrounding induration, with the longest diameter of 26 mm, was observed on the right side of the patient's tongue (Figure 3A,B).

**TABLE 1:** Blood examination on first visit.

Blood cell count		Blood chemistry	
White blood cell	6020/*μ*L	Total protein	7.3 g/dL
Neutrocyte	3230/*μ*L	Albumin	4.2 g/dL
Lymphocyte	2170/*μ*L	Sodium	144 mEq/L
Monocyte	220/*μ*L	Potassium	4.3 mEq/L
Eosinocyte	230/*μ*L	Chloride	108 mEq/L
Basocyte	60/*μ*L	Blood urea nitrogen	18.9 mg/dL
Red blood cell	443 × 10^4^/*μ*L	Creatinine	1.01 mg/dL
Hemoglobin	14.5 g/dL	Asoartate aminotransferase	22 IU/L
Hematocrit	43.9%	Alanine aminotransferase	26 IU/L
MCV	99.0 fL ↑	γ‐glutamyl transpeptidase	27 IU/L
MCH	32.7 pg	Total bilirbin	0.4 mg/dL
MCHC	33.0 g/dL	Direct bilirbin	0.1 mg/dL
Platelet	25.4 × 10^4^ /*μ*L	Indirect bilirbin	0.3 mg/dL
		Alkaline phosphatase	64 IU/L
		C‐reactive protein	0.15 mg/dL ↑

Abbreviations: MCV, mean corpuscular volume; MCH, mean corpuscular hemoglobin; MCHC, mean corpuscular hemoglobin concentration; ↑, value higher than the standard value.

The tongue tumor was biopsied and diagnosed as well‐differentiated squamous cell carcinoma histopathologically. CT revealed a 24.0 × 19.3 × 14.2‐mm, contrast‐enhanced tumor extending from the right side of the tongue (Figure 2B). Hypo‐absorption of contrast was seen in the left level IB and III lymph nodes. Magnetic resonance imaging revealed a contrast‐enhanced 23.4 × 19.6‐mm, tongue tumor with a depth of invasion (DOI) of 13.9 mm (data not shown). FDG‐PET/CT revealed a tumor with abnormal accumulation and with a maximum standardized uptake value (SUV_max_) of 14.5 at the right lingual border. Additionally, enlarged lymph nodes were seen at left level IB and III, with abnormal accumulation and SUV_max_ values of 4.10 and 9.00, respectively (Figure 3C). Although these preoperative imaging findings of the lymph nodes at left level IB and III suggested malignant tumors, histopathology could not confirm whether the changes in these lymph nodes indicated metastases of the tongue cancer or recurrence of HL. Therefore, our preoperative diagnosis was tongue cancer with cervical lymph node metastasis (cT3N2cM0), or tongue cancer (cT3N0M0) and recurrent HL.

Surgery was performed on the basis of the results of a biopsy of an enlarged lymph node at left level IB. As this lymph node was identified as metastatic squamous cell carcinoma on intraoperative frozen section rapid pathological diagnosis, bilateral neck dissection was performed.

**FIGURE 4:**
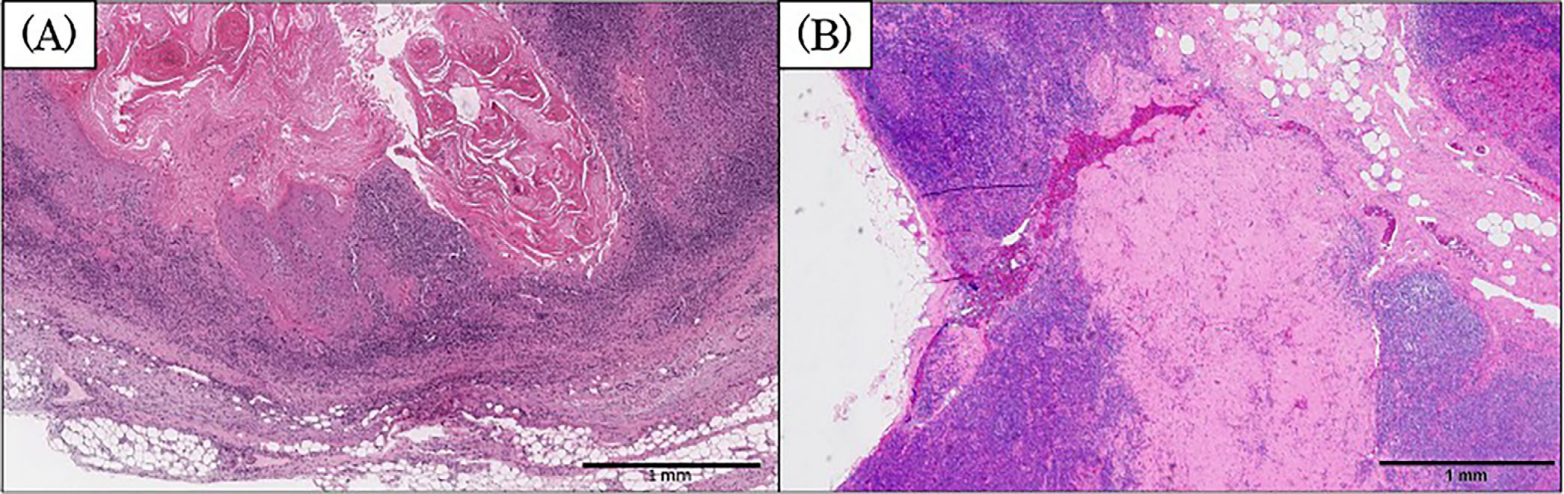
Histopathological findings of the lymph node removed during neck dissection. Level III lymph node on the left side of the neck (A). Metastasis of squamous cell carcinoma is visible within the lymph node. There is no obvious extranodal extension (×40). The right cervical lymph node (B). This lymph node shows no normal lymph node structure, with internal vitreous degeneration and no evidence of squamous cell carcinoma metastasis or Hodgkin lymphoma (×40).

Postoperative histopathological examination revealed that the tongue cancer was radically resected, and one metastatic lymph node of squamous cell carcinoma at left level IB and three nodes at left level III were found, none of which showed extranodal extension (Figure 4A). Additionally, the right cervical lymph node, which was in remission after HL treatment, did not show the structures seen in normal lymph nodes, such as lymph follicles, cortex, and medullary cords. Instead, internal vitreous degeneration was seen, with no evidence of squamous cell carcinoma metastasis or HL (Figure 4B). Postoperative radiation therapy with cisplatin chemotherapy was proposed owing to the multiple cervical lymph node metastases of tongue cancer. However, in accordance with the patient's strong desire, a follow‐up plan was chosen. One year and 7 months after the tongue cancer treatment, there was no evidence of metastasis of the tongue cancer or recurrence of new Hodgkin‐affected lymph nodes.

## 3 DISCUSSION

Determining the efficacy of treatment for hematological malignancies is difficult to evaluate pathologically, as is the case with solid tumors. In this case, the patient developed cervical lymph metastasis of tongue cancer, which allowed incidental pathological evaluation of the condition of the lymph nodes in which HL was in remission. We found no reports showing the pathology of lymph nodes with HL in remission, which is rare; pathological confirmation is usually obtained incidentally during autopsy. Usually, the determination of treatment efficacy for HL is as follows: To determine the efficacy of initial therapy after treatment for HL, the Deauville criteria, based on visual evaluation of FDG‐PET/CT, were defined in 2009.^3^, ^4^ These criteria are based on a score of 1–3 (negative) and 4–5 (positive). In this case, there was no FDG accumulation in the right cervical lymph nodes at the patient's initial visit to our department, and the therapeutic effect was Deauville 1 or 2 points. When determining the efficacy of HL treatment, it is impossible to remove all diseased lymph nodes and observe them pathologically because of the physical invasiveness to the patient, and imaging evaluation is the mainstay of the process.

In this case, the patient had squamous cell carcinoma on the right side of the tongue, and imaging studies suspected metastasis of tongue cancer or HL recurrence in the cervical lymph nodes on the left side. The tongue cancer in this case was located at the apex of the tongue, with a depth of invasion (DOI)of 13.9 mm. In the cervical lymph nodes, there were metastases only on the contralateral side and none on the affected side. The lymphatic vessels in the lingual apex penetrate the mylohyoid muscle to the submandibular lymph nodes; those in the lateral border of the tongue penetrate the mylohyoid muscle to the submandibular lymph nodes; and those in the central tongue and posterior tongue reach the internal deep neck lymph nodes through the surface and back of the glossohyoid muscle.^5^, ^6^ As the tongue cancer in this case was located at the apex of the tongue, and the DOI was deep, it is possible that the lymphatic flow pathways could have traveled to the neck bilaterally, either superficially or deeply. As the *T* classification of the patient's tongue cancer was cT3, the treatment plan was to perform prophylactic right‐sided neck dissection. After the rapid intraoperative pathological confirmation of the diagnosis, bilateral neck dissection was performed. If HL had been detected in the left cervical lymph nodes, only right‐sided neck dissection would have been performed.

Histopathologically, the cervical lymph nodes on the affected side in our case showed no normal lymph node structure. The interior part of these lymph nodes showed vitreous degeneration, which suggested that lymphatic flow may have been blocked; thus, no lymph node metastasis was seen. It is likely that the previous chemotherapy for HL, which involved a primary lesion in the right cervical lymph nodes, caused degeneration of the lesion and blocked normal lymph flow. Fortunately, in this case, we were incidentally able to explore and examine the histopathological findings after remission of HL. These data may be valuable because to our knowledge, there have been no previous reports showing the pathological findings or evaluation of foci that have achieved remission status with chemotherapy for HL. There have been reports of cases of recurrent HL controlled with intense chemotherapy, confirmed by FDG‐PET/CT.^7^ The usefulness of biomarkers, such as thymus and activation‐regulated chemokine as prognostic and treatment monitoring for HL has also been reported.^8^, ^9^ We hope that this report of the pathology after HL treatment will contribute to the correlation of the pathological findings of the lymph nodes of remission cases and image monitoring, such as with FDG‐PET/CT, in the future.

## 4 CONCLUSION

In this case, because of the development of metastatic cervical lymph nodes from the tongue cancer, we performed dissection of the neck (in which the cervical lymph nodes were the main lesion) after treatment for HL. This allowed for histopathological identification of a lymph node that lost all normal structure after treatment for HL. This is a valuable case report demonstrating changes in the lymph node structure after HL treatment. As post‐treatment evaluation of HL is performed only by imaging evaluation with FDG‐PET/CT, this case provides valuable clinical data showing the histopathological findings after HL treatment.

## AUTHOR CONTRIBUTIONS

Yu Ohashi contributed to the study conception and design and wrote the manuscript. Kiyoto Shiga, Hiroyuki Yamada, Katsunori Katagiri, Daisuke Saito, Shin‐ichi Oikawa, Kodai Tsuchida, Jun Miyaguchi, Takahiro Kusaka contributed to data acquisition. All authors have read and approved the final version of this manuscript.

## FUNDING INFORMATION

This case report was funded by JSPS KAKENHI (Grant Number JP20K10251).

## CONFLICT OF INTEREST STATEMENT

The authors have stated explicitly that there are no conflicts of interest in connection with this article.

## ETHICS STATEMENT

The Iwate Medical University Institutional Review Board exempted ethics approval for case reports.

## INFORMED CONSENT

Full consent for participation and publication was provided by the patient.

## Data Availability

The data that support the findings of this study are available from the corresponding author upon reasonable request.
